# Gibbs energy functions with the vacancy complexes in the Al-Cu binary system

**DOI:** 10.1016/j.dib.2018.09.092

**Published:** 2018-10-12

**Authors:** Taichi Abe, Masato Shimono, Kiyoshi Hashimoto, Cenk Kocer

**Affiliations:** a Research and Services Division of Materials Data and Integrated System (MaDIS), National Institute for Materials Science, 1-2-1 Sengen, Tsukuba, Ibaraki 305-0047 Japan; b Research Center for Structural Materials, National Institute for Materials Science, Japan; c School of Physics, The University of Sydney, Australia

## Abstract

The Gibbs energy functions of the phases in the Al-Cu binary system are taken from the CALPHAD-type thermodynamic assessment (Witusiewicz et al., 2004; Ansara et al., 1998) [1], [2], where the effect of the monovacancy (Va), divacancy (VaVa) and Va-solute atom pair are taken into account based on the formulation (Abe et al., In press). The divacancy is modeled as an associate, VaVa, in the FCC solid solution. The contributions from the Va-solute pair are included through the ternary excess Gibbs energy term. Using the Gibbs energy functions provided in this data article, the fractions of the monovacancies and divacancies, even in various metastable conditions, can be calculated. Since the Gibbs energy functions and phase descriptions are written in the TDB (Thermodynamic DataBase) format, one can use this file with various thermodynamic software packages, such as OpenCalphad [3] etc.

**Specifications table**

**Table:** 

Subject area	*Physics*
More specific subject area	*Computational thermodynamics*
Type of data	*Tables, figures, and a TDB file*
How data was acquired	*Gibbs energy functions are taken from the published paper. For FCC phase vacancies are described based on our work.*
Data format	*Assessed*
Experimental factors	*N.A.*
Experimental features	*N.A.*
Data source location	*Tuskuba, Ibaraki, Japan*
Data accessibility	*Data are available here with this article*

**Value of the data**

• Using thermodynamic calculation software packages one can calculate phase equilibria in the Al-Cu binary system where the effects of monovacancy, divacancy and vacancy-solute atom pairs in the FCC phase are included.
• Even in metastable states, such as a supersaturated FCC solid solution, vacancy fractions can be estimated.
• Even in metastable states, such as higher vacancy fractions after heavy deformation or irradiation, it is possible to estimate the properties of the monovacancy and the vacancy complexes.

## 1 Data

This includes experimentally measured thermodynamic properties of vacancies (Section 2) and Gibbs energies of phase with vacancies for thermodynamic calculations on software packages [1], [2], [3] in the TDB format (Section 3). Thermodynamic models and descriptions [4] are briefly explained in Section 1.

### 1.1 Monovacancy

The monovacancy (Va) was introduced to a substitutional solution model as a non-preserved quantity [5], where the Gibbs energy of the monovacancy formation was described in the regular term, LA,Va(0)=HAVaf−SAVafT−GmVa, and the Gibbs energy of the empty endmember, $G_{m}^{Va}$, was set +10*RT* to avoid an unwilling miscibility gap at very high temperatures. The vacancy formation entropy, SAVaf, and enthalpy, HAVaf, in the regular term were optimized so as to reproduce experimental data with reasonable accuracy.

### 1.2 Divacancy

The divacancy, which is defined as a pair of vacancies in the nearest neighbor distance, was treated as an associate, VaVa, using the associate solution model [3], [6], [7], [8] where the Gibbs energy of the divacancy formation in matrix A was described in the regular term, $L_{A,VaVa}^{\left(0\right)}$, and the Gibbs energy of the empty associate, $G_{m}^{VaVa}$, was set +10*RT*. Using the binding entropy, $S_{A}^{Bind-VaVa}$, and enthalpy, $H_{A}^{Bind-VaVa}$ , and monovacancy formation entropy and enthalpy defined above, the divacancy formation entropy and enthalpy in the FCC lattice can be given as SAAssociatef=Rln(6)+2SAVaf+SABind-VaVa and HAAssociatef=2HAVaf+HABind-VaVa in the regular term, and were optimized so as to reproduce experimental data with reasonable accuracy.

### 1.3 Vacancy-solute atom pair

The effect of the vacancy-solute atom pair, which is defined as a pair of a monovacancy and a solute atom within the nearest neighbor distance, was considered using the ternary excess Gibbs energy term as LA,B,Va0=12HAB-Va_Bind where $H_{A}^{B-Va\_Bind}$ is the binding enthalpy between the monovacancy and a solute atom B in matrix A. This relation was obtained from the comparison between parameters in the Lomer model [9] where the binding energy is considered and in the substitutional solution model using the Redlich-Kister polynomial [10].

## 2 Properties of vacancies

### 2.1 Pure Al

**Fig. 1:**
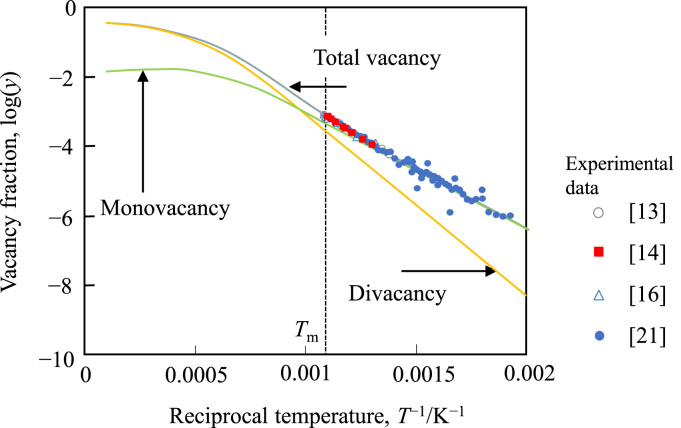
Temperature dependency of the vacancy fractions in pure Al. Plots are experimental data and solid lines are calculated results using the TDB file given in Table 4.

The properties of vacancies in Al in literature are summarized in Table 1. The calculated vacancy fractions in pure Al are presented in Fig. 1 with experimental data [13], [14], [16], [21].

**Table 1:** Properties of vacancies in pure Al where symbols denote as Cp: specific heat, DD: differential dilatometry, PA: positron annihilation, RR: resistivity measurements, *: divacancy binding enthalpy, **: divacancy formation entropy, and ***: calculated value in this work.

Method	Monovacancy formation enthalpy, eV	Monovacancy formation entropy	Total vacancy fraction at the melting point	Reference	Method	Monovacancy formation enthalpy, eV	Monovacancy formation entropy	Total vacancy fraction at the melting point	Reference
Cp	0.79	2.9***	1.03 × 10^−3^	[11]	PA	0.68			[28]
Cp	0.7	1.3***	6 × 10^−4^	[12]	PA	0.66	1.3	1.0 × 10^−3^***	[29]
DD	0.76	2.4	9.4 × 10^−4^	[13]	PA	1.02*	5.4**		[29]
DD	0.66	0.6	9.8 × 10^−4^	[14]	PA	0.71			[30]
DD	0.23*	2.2**		[14]	PA	0.66			[31]
DD	0.77	1.5 ***	3 × 10^−4^	[15]	PA	0.66			[32]
DD	0.71	1.76	8.5 × 10^−4^***	[16]	PA	0.675			[33]
DD			8.70 × 10^−4^	[17]	PA	0.685			[33]
DD	0.65	1.1 ***	9 × 10^−4^	[18]	PA	0.69			[34]
DD	0.64	1.2***	1.1 × 10^−3^	[19]	PA	0.68			[35]
DD	0.67	2.66	3.4 × 10^−3^	[20]	PA	0.66			[36]
DD	0.67	1.1	7.2 × 10^−4^***	[21]	PA	0.66	1.28	9.8 × 10^−4^***	[37]
DD	0.20*	0.7**		[21]	PA	0.30*	1.24**		[37]
PA	0.68			[18]	RR	0.73			[38]
PA	0.65			[18]	RR	0.69			[39]
PA	0.6			[18]	RR	0.66			[40]
PA	0.67			[22]	RR	0.702	1.69	8.9 × 10^−4^***	[41]
PA	0.62			[23]	RR	0.76			[42]
PA	0.64			[24]	RR	0.76	2.1	6 × 10^−4^	[43]
PA	0.66			[25]	RR	0.74			[44]
PA	0.68			[26]	RR	0.65	0.78	6.7 × 10^−4^***	[45]
PA	0.63			[27]	RR	0.17*			[45]
PA	0.64			[27]	RR	0.17*			[46]

### 2.2 Pure Cu

**Fig. 2:**
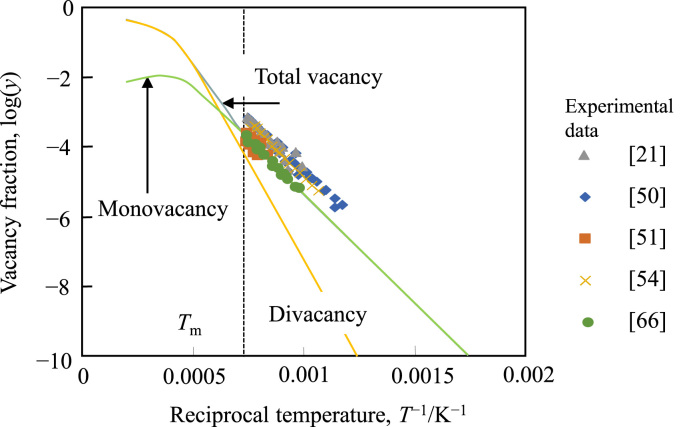
Temperature dependency of the vacancy fractions in pure Cu. Plots are experimental data and solid lines are calculated results using the TDB file given in Table 4.

The properties of vacancies in Cu in literature are summarized in Table 2. The calculated vacancy fractions in pure Cu are presented in Fig. 2 with experimental data [21], [49], [50], [53], [65].

**Table 2:** Properties of vacancies in pure Cu where symbols denote Cp: specific heat, DD: differential dilatometry, PA: positron annihilation, RR: resistivity measurements, *: divacancy binding enthalpy, **: divacancy formation entropy, and ***: calculated value in this work.

Method	Monovacancy formation enthalpy, eV	Monovacancy formation entropy	Total vacancy fraction at the melting point	Reference	Method	Monovacancy formation enthalpy, eV	Monovacancy formation entropy	Total vacancy fraction at the melting point	Reference
Cp	1.05	3.69	5.0 × 10^−3^***	[47]	PA	1.13			[61]
DD	1.18	1.6	2.1 × 10^−4^	[48]	PA	1.28			[61]
DD	1.19	3	7.6 × 10^−4^	[49]	PA	1.2			[62]
DD	1.17	1.5	2.0 × 10^−4^	[50]	PA	1.42			[63]
PA	1.26			[51]	PA	0.98			[64]
PA	1.28			[52]	PA	1.19			[21]
PA	0.3*			[52]	RR	1.27			[65]
PA	1.21			[52]	RR	1.3	2.8	2.4 × 10^−4^***	[66]
PA	0.15*			[52]	RR	1.06	−0.3	1.6 × 10^−4^ ***	[67]
PA	1.29			[53]	RR	0.92			[68]
PA	1.17			[54]	RR	1	2.0***	1.5 × 10^−3^	[69]
PA	1.28			[55]	RR	1.14			[70]
PA	1.22			[56]	RR	1		4.00 × 10^−4^	[71]
PA	1.19			[56]	RR	0.45*			[71]
PA	1.04			[56]	RR	1.03	1.4	6.0 × 10^−4^***	[72]
PA	1.22			[57]	RR	0.20*	3.5**		[72]
PA	1.33			[58]	RR	0.9			[73]
PA	1.31			[58]	RR	1.03	0.3	2.0 × 10^−4^ ***	[74]
PA	1.29			[59]	RR	0.54*	2**		[74]
PA	1.16	1.3	1.8 × 10^−4^***	[60]					
PA	0.32*	3.7**		[60]

### 2.3 Al-Cu FCC solid solution

The calculated vacancy fraction in an FCC solid solution in the Al-Cu binary system is presented in Ref. [4]. The properties of the vacancies in the FCC solid solution phase are listed in Table 3.

**Table 3:** Properties of vacancies in the Al-Cu solid solution where symbols denote DD: Differential Dilatometry, TEM: Transmission electron microscopy, PA: positron annihilation, RR: Resistivity measurements, and *: divacancy formation enthalpy, eV.

Composition, at.%	Method	Monovacancy formation enthalpy, eV	Va-Solute binding energy, eV	Va-Solute binding entropy	Total vacancy fraction	Reference
Al-0.41Cu	DD			0		[75]
Al-0.45Cu	TEM				3 × 10^−5^ (813 K)	[76]
Al-0.45Cu	TEM				5 × 10^−3^ (873 K)	[76]
Al-0.86Cu	TEM				1× 10^−5^ (813 K)	[76]
Al-0.86Cu	TEM				1 × 10^−3^ (873 K)	[76]
Al-1.7Cu	TEM				3 × 10^−5^ (813 K)	[76]
Al-0.73Cu	RR	0.77	0.2			[77]
Cu-2.0Al	PA	1.17				[78]
Cu-2.0Al	PA	2.30*				[78]
Cu-8.5Al	PA	1.06				[78]
Cu-13.7Al	PA	1				[78]
Cu-13.7Al	PA	0.99				[78]
Cu-16.6Al	PA	0.93				[78]
Cu-0.5Al	PA		0.17			[79]

## 3 TDB file for the thermodynamic calculations

The TDB file for the FCC phase with vacancies are listed in Table 4 where the parameters of vacancies in Table 2 of Ref. [3] are written in the TDB format [80]. This TDB file can be used with various software packages [5]. The full TDB file for the Al-Cu binary system is given as a supplement.

**Table 4:** The TDB file for the parameters listed in Table 2 of Ref. [3]. The full TDB file for the Al-Cu binary system is provided as a supplement.

SPECIES VAVA VA2!		
FUNCTION RR	300 +8.3145;	6000 N!
FUNCTION ZZ	300 +12;	6000 N!
FUNCTION EVJ	300 +96485;	6000 N!
FUNCTION HVAL	300 +0.66*EVJ;	6000 N!
FUNCTION HVCU	300 +1.23*EVJ;	6000 N!
FUNCTION SVAL	300 +0.70;	6000 N!
FUNCTION SVCU	300 +1.87;	6000 N!
FUNCTION HVVAL	300 -0.28*EVJ+2*HVAL;	6000 N!
FUNCTION HVVCU	300 -0.23*EVJ+2*HVCU;	6000 N!
FUNCTION SVVAL	300 +LN(6)+1.2+2*SVAL;	6000 N!
FUNCTION SVVCU	300 +LN(6)+2.8+2*SVCU;	6000 N!
FUNCTION BCUVA	300 -0.00*EVJ;	6000 N!
FUNCTION BALVA	300 -0.15*EVJ;	6000 N!
PHASE FCC % 1 1 !		
CONSTITUENT FCC : AL,CU,VA,VaVa: !		
PARA G(FCC,AL;0)	300 +GHSERAL;	2900 N!
PARA G(FCC,CU;0)	300 +GHSERCU;	3200 N!
PARA G(FCC,AL,CU;0)	300 -53520+2*T;	3200 N!
PARA G(FCC,AL,CU;1)	300 +38590-2*T;	3200 N!
PARA G(FCC,AL,CU;2)	300 +1170;	3200 N!
PARA G(FCC,VA;0)	300 +10*RR*T;	6000 N!
PARA G(FCC,VAVA;0)	300 +10*RR*T;	6000 N!
PARA G(FCC,AL,VAVA;0)	300 +HVVAL-SVVAL*RR*T-10*RR*T;	6000 N!
PARA G(FCC,CU,VAVA;0)	300 +HVVCU-SVVCU*RR*T-10*RR*T;	6000 N!
PARA G(FCC,AL,VA;0)	300 +HVAL -SVAL*RR*T -10*RR*T;	6000 N!
PARA G(FCC,CU,VA;0)	300 +HVCU -SVCU*RR*T -10*RR*T;	6000 N!
PARA G(FCC,AL,CU,VA;0)	300 +ZZ*BCUVA;	6000 N!
PARA G(FCC,AL,CU,VA;1)	300 +ZZ*BALVA;	6000 N!
PARA G(FCC,AL,CU,VA;2)	300 +0;	6000 N!
