# Supplementary material for: Gibbs energy functions with the vacancy complexes in the Al-Cu binary system
Source: Data Brief. 2018 Oct 12;21:432–40. doi: 10.1016/j.dib.2018.09.092 (PMC6198129; doi:10.1016/j.dib.2018.09.092)
Supplement: Supplementary file 2 — Supplementary material [file mmc2.doc]

$Al-Cu

$

$ TDB-file for the thermodynamic assessment of the Al-Cu system with VA AND VV.

$

$-----------------------------------------------------------------------------

$ 2018.6.13

$

$ TDB file created by T.Abe and K.Hashimoto

$

$ National Institute for Materials Science (NIMS)

$ 1-2-1 Sengen, Tsukuba, Ibaraki 305-0047, Japan

$ e-mail: abe.taichi(at)nims.go.jp

$ Copyright (C) NIMS 2018

$

$ ------------------------------------------------------------------------------

$ PARAMETERS ARE TAKEN FROM

$

$ The Ag-Al-Cu system Part I: Reassessment of the constituent binaries

$ on the basis of new experimental data.

$ V.T. Witusiewicz, U. Hecht, S.G. Fries, S. Rex,

$ Journal of Alloys and Compounds, 385 (2004), 133-143.

$

$ ------------------------------------------------------------------------------

$ Commnets

$ The fcc phase is written by the associate solution model where the Va-Va

$ complex (associate) is defined.

$ T.A.

$ ------------------------------------------------------------------------------

ELEMENT /- ELECTRON_GAS 0.0000E+00 0.0000E+00 0.0000E+00!

ELEMENT VA VACUUM 0.0000E+00 0.0000E+00 0.0000E+00!

ELEMENT AL FCC_A1 2.6982E+01 4.5773E+03 2.8322E+01!

ELEMENT CU FCC_A1 6.3546E+01 5.0041E+03 3.3150E+01!

SPECIES VV VA2!

$--------1---------2---------3---------4---------5---------6---------7---------8

$

$ FUNCTION AL 91DIN

$ -------------------------------------

FUNCTION GHSERAL 298.15

-7976.15+137.093038*T-24.3671976*T*LN(T)-1.884662E-3*T**2-0.877664E-6*T**3

+74092*T**(-1); 700.00 Y

-11276.24+223.048446*T-38.5844296*T*LN(T)+18.531982E-3*T**2

-5.764227E-6*T**3+74092*T**(-1); 933.47 Y

-11278.378+188.684153*T-31.748192*T*LN(T)-1230.524E25*T**(-9);

2900.00 N !

FUNCTION GLIQAL 298.15

+3028.879+125.251171*T-24.3671976*T*LN(T)-1.884662E-3*T**2-0.877664E-6*T**3

+74092*T**(-1)+79.337E-21*T**7; 700.00 Y

-271.21+211.206579*T-38.5844296*T*LN(T)+18.531982E-3*T**2-5.764227E-6*T**3

+74092*T**(-1)+79.337E-21*T**7; 933.47 Y

-795.996+177.430178*T-31.748192*T*LN(T); 2900.00 N !

FUNCTION GBCCAL 298.15 +10083-4.813*T+GHSERAL; 6000 N !

FUNCTION GALLAV 298.15 +15000+3*GHSERAL; 3000 N !

$ -------------------------------------

$ FUNCTION CU 91DIN

$ -------------------------------------

FUNCTION GHSERCU 298.15

-7770.458+130.485235*T-24.112392*T*LN(T)-2.65684E-3*T**2+0.129223E-6*T**3

+52478*T**(-1); 1357.77 Y

-13542.026+183.803828*T-31.38*T*LN(T)+364.2E27*T**(-9); 3200.00 N !

FUNCTION GLIQCU 298.15

+5194.277+120.973331*T-24.112392*T*LN(T)-2.65684E-3*T**2+0.129223E-6*T**3

+52478*T**(-1)-584.9E-23*T**7; 1357.77 Y

-46.545+173.881484*T-31.38*T*LN(T); 3200.00 N !

FUNCTION GBCCCU 298.15

-3753.458+129.230235*T-24.112392*T*LN(T)-2.65684E-3*T**2+0.129223E-6*T**3

+52478*T**(-1); 1357.77 Y

-9525.026+182.548828*T-31.38*T*LN(T)+364.167E27*T**(-9); 3200.00 N !

FUNCTION GHCPCU 298.15

-7170.458+130.685235*T-24.112392*T*LN(T)-2.65684E-3*T**2+0.129223E-6*T**3

+52478*T**(-1); 1357.77 Y

-12942.026+184.003828*T-31.38*T*LN(T)+364.167E27*T**(-9); 3200.00 N !

$------------------------------------------------------------------------------

TYPE_DEFINITION % SEQ *!

DEFINE_SYSTEM_DEFAULT ELEMENT 2 !

DEFAULT_COMMAND DEF_SYS_ELEMENT VA /- !

$------------------------------------------------------------------------------

$ PARAMETERS FOR LIQUID PHASE

$------------------------------------------------------------------------------

PHASE LIQUID % 1 1 !

CONSTITUENT LIQUID : AL,CU : !

PARAMETER G(LIQUID,AL;0) 298.15 +GLIQAL; 2900 N !

PARAMETER G(LIQUID,CU;0) 298.15 +GLIQCU; 3200 N !

$ Witusiewicz2004

PARAMETER G(LIQUID,AL,CU;0) 298.15 -67094+8.555*T; 3200 N !

PARAMETER G(LIQUID,AL,CU;1) 298.15 +32148-7.118*T; 3200 N !

PARAMETER G(LIQUID,AL,CU;2) 298.15 +5915-5.889*T; 3200 N !

PARAMETER G(LIQUID,AL,CU;3) 298.15 -8175+6.049*T; 3200 N !

$------------------------------------------------------------------------------

$ PARAMETERS FOR BCC PHASE

$------------------------------------------------------------------------------

PHASE BCC % 1 1 !

CONSTITUENT BCC : AL,CU : !

PARAMETER G(BCC,AL;0) 298.15 +10083-4.813*T+GHSERAL; 3000 N !

PARAMETER G(BCC,CU;0) 298.15 +GBCCCU; 3200 N !

$ Saunders,1991, COST database

PARAMETER G(BCC,AL,CU;0) 298.15 -73554+4.0*T; 3200 N !

PARAMETER G(BCC,AL,CU;1) 298.15 +51500-11.84*T; 3200 N !

$------------------------------------------------------------------------------

$ FCC: AL-CU-VA PARAMETERS, Associate solution model

$------------------------------------------------------------------------------

FUNCTION RR 300 +8.3145; 6000 N !

FUNCTION ZZ 300 +12; 6000 N !

FUNCTION EVJ 300 +96485; 6000 N !

$MONO-VACANCY

FUNCTION HVAL 300 +0.66*EVJ; 6000 N !

FUNCTION HVCU 300 +1.23*EVJ; 6000 N !

FUNCTION SVAL 300 +0.70; 6000 N !

FUNCTION SVCU 300 +1.87; 6000 N !

$DIVACANCY

FUNCTION HVVAL 300 -0.28*EVJ+2*HVAL; 6000 N !

FUNCTION HVVCU 300 -0.23*EVJ+2*HVCU; 6000 N !

FUNCTION SVVAL 300 +LN(6)+1.2+2*SVAL; 6000 N !

FUNCTION SVVCU 300 +LN(6)+2.8+2*SVCU; 6000 N !

$SOLUTE-VA PAIRS

FUNCTION BCUVA 300 -0.00*EVJ; 6000 N !

FUNCTION BALVA 300 -0.15*EVJ; 6000 N !

$------------------------------------------------------------------------------

PHASE FCC % 1 1 !

CONSTITUENT FCC : AL,CU,VA,VV: !

PARAMETER G(FCC,AL;0) 300 GHSERAL; 2900 N !

PARAMETER G(FCC,CU;0) 300 GHSERCU; 3200 N !

$ Saunders,1991 ,COST database

PARAMETER G(FCC,AL,CU;0) 300 -53520+2*T; 3200 N !

PARAMETER G(FCC,AL,CU;1) 300 +38590-2*T; 3200 N !

PARAMETER G(FCC,AL,CU;2) 300 +1170; 3200 N !

$Parameters for the vacancies

PARAMETER G(FCC,VA;0) 300 +10*RR*T; 6000 N !

PARAMETER G(FCC,VV;0) 300 +10*RR*T; 6000 N !

PARAMETER G(FCC,AL,VV;0) 300 +HVVAL-SVVAL*RR*T-10*RR*T; 6000 N !

PARAMETER G(FCC,CU,VV;0) 300 +HVVCU-SVVCU*RR*T-10*RR*T; 6000 N !

PARAMETER G(FCC,AL,VA;0) 300 +HVAL -SVAL*RR*T -10*RR*T; 6000 N !

PARAMETER G(FCC,CU,VA;0) 300 +HVCU -SVCU*RR*T -10*RR*T; 6000 N !

PARAMETER G(FCC,AL,CU,VA;0) 300 +ZZ*BCUVA; 6000 N !

PARAMETER G(FCC,AL,CU,VA;1) 300 +ZZ*BALVA; 6000 N !

PARAMETER G(FCC,AL,CU,VA;2) 300 +0; 6000 N !

$------------------------------------------------------------------------------

$ PARAMETERS FOR HCP PHASE

$------------------------------------------------------------------------------

PHASE HCP % 1 1 !

CONSTITUENT HCP : AL,CU : !

PARAMETER G(HCP,AL;0) 298.15 +5481-1.8*T+GHSERAL; 3000 N !

PARAMETER G(HCP,CU;0) 298.15 GHCPCU; 3200 N !

$------------------------------------------------------------------------------

$ PARAMETERS FOR AL-CU INTERMETALLIC COMPOUNDS (Saunders,1991)

$------------------------------------------------------------------------------

PHASE ALCU_DELTA % 2 2 3 !

CONSTITUENT ALCU_DELTA : AL : CU : !

PARAMETER G(ALCU_DELTA,AL:CU;0) 298.15 -106700.0+3.0*T

+2*GHSERAL+3*GHSERCU; 3200 N !

PHASE ALCU_EPSILON % 2 1 1 !

CONSTITUENT ALCU_EPSILON : AL,CU : CU : !

PARAMETER G(ALCU_EPSILON,AL:CU;0) 298.15 -36976+1.2*T

+GHSERAL+GHSERCU; 3200 N !

PARAMETER G(ALCU_EPSILON,CU:CU;0) 298.15 +2*GBCCCU; 3200 N !

PARAMETER G(ALCU_EPSILON,AL,CU:CU;0) 298.15 +7600-24*T; 3200 N !

PARAMETER G(ALCU_EPSILON,AL,CU:CU;1) 298.15 -72000; 3200 N !

PHASE ALCU_ETA % 2 1 1 !

CONSTITUENT ALCU_ETA : AL,CU : CU : !

PARAMETER G(ALCU_ETA,AL:CU;0) 298.15 -40560.0+3.14*T

+GHSERAL+GHSERCU; 3200 N !

PARAMETER G(ALCU_ETA,CU:CU;0) 298.15 +2*GBCCCU; 3200 N !

PARAMETER G(ALCU_ETA,AL,CU:CU;0) 298.15 -25740-20*T; 3200 N !

PHASE ALCU_THETA % 2 2 1 !

CONSTITUENT ALCU_THETA : AL : AL,CU : !

PARAMETER G(ALCU_THETA,AL:AL;0) 298.15 +3*GBCCAL; 3200 N !

PARAMETER G(ALCU_THETA,AL:CU;0) 298.15 -47406.0+6.75*T

+2*GHSERAL+GHSERCU; 3200 N !

PARAMETER G(ALCU_THETA,AL:AL,CU;0) 298.15 +2211; 3200 N !

PHASE ALCU_ZETA % 2 9 11 !

CONSTITUENT ALCU_ZETA : AL : CU : !

PARAMETER G(ALCU_ZETA,AL:CU;0) 298.15 -420000.0+18.0*T

+9*GHSERAL+11*GHSERCU; 3200 N !

$ Witusiewicz.2004-----------------

PHASE ALCU_GAMMA_D83 % 3 4 1 8 !

CONSTITUENT ALCU_GAMMA_D83 : AL : AL,CU : CU : !

PARAMETER G(ALCU_GAMMA_D83,AL:AL:CU;0) 298.15 -277739+215*T-30*T*LN(T)

+5*GHSERAL+8*GHSERCU; 3200 N !

PARAMETER G(ALCU_GAMMA_D83,AL:CU:CU;0) 298.15 -280501+379.6*T-52*T*LN(T)

+4*GHSERAL+9*GHSERCU; 3200 N !

$----------------------------------

PHASE ALCU_GAMMA_H % 3 4 1 8 !

CONSTITUENT ALCU_GAMMA_H : AL : AL,CU : CU : !

PARAMETER G(ALCU_GAMMA_H,AL:AL:CU;0) 298.15 -219258.0-45.5*T

+5*GHSERAL+8*GHSERCU; 3200 N !

PARAMETER G(ALCU_GAMMA_H,AL:CU:CU;0) 298.15 -200460.0-58.5*T

+4*GHSERAL+9*GHSERCU; 3200 N !

PHASE LAVES_C14 % 2 2 1 !

CONSTITUENT LAVES_C14 : AL,CU : AL,CU : !

PARAMETER G(LAVES_C14,AL:AL;0) 298.15 +15000+3*GHSERAL; 3200 N !

PARAMETER G(LAVES_C14,AL:CU;0) 298.15 +15000+2*GHSERAL+GHSERCU; 3200 N !

PARAMETER G(LAVES_C14,CU:AL;0) 298.15 +15000+GHSERAL+2*GHSERCU; 3200 N !

PARAMETER G(LAVES_C14,CU:CU;0) 298.15 +15000+3*GHSERCU; 3200 N !

PARAMETER G(LAVES_C14,AL,CU:AL;0) 298.15 -24000+2.44*T; 3200 N !

PARAMETER G(LAVES_C14,AL,CU:CU;0) 298.15 -24000+2.44*T; 3200 N !

PHASE LAVES_C15 % 2 2 1 !

CONSTITUENT LAVES_C15 : AL,CU : AL,CU : !

PARAMETER G(LAVES_C15,AL:AL;0) 298.15 +15000+3*GHSERAL; 3200 N !

PARAMETER G(LAVES_C15,AL:CU;0) 298.15 +15000+2*GHSERAL+GHSERCU; 3200 N !

PARAMETER G(LAVES_C15,CU:AL;0) 298.15 +15000+GHSERAL+2*GHSERCU; 3200 N !

PARAMETER G(LAVES_C15,CU:CU;0) 298.15 +15000+3*GHSERCU; 3200 N !

PARAMETER G(LAVES_C15,AL,CU:AL;0) 298.15 -105000+1.615*T; 3200 N !

PARAMETER G(LAVES_C15,AL,CU:CU;0) 298.15 -105000+1.615*T; 3200 N !

PHASE LAVES_C36 % 2 2 1 !

CONSTITUENT LAVES_C36 : AL,CU : AL,CU : !

PARAMETER G(LAVES_C36,AL:AL;0) 298.15 +15000+3*GHSERAL; 3200 N !

PARAMETER G(LAVES_C36,AL:CU;0) 298.15 +15000+2*GHSERAL+GHSERCU; 3200 N !

PARAMETER G(LAVES_C36,CU:AL;0) 298.15 +15000+GHSERAL+2*GHSERCU; 3200 N !

PARAMETER G(LAVES_C36,CU:CU;0) 298.15 +15000+3*GHSERCU; 3200 N !

PARAMETER G(LAVES_C36,AL,CU:AL;0) 298.15 -126169+14.61*T; 3200 N !

PARAMETER G(LAVES_C36,AL,CU:CU;0) 298.15 -126169+14.61*T; 3200 N !

$------------------------------------------------------------------------------

$end
